# Representative Pores: An Efficient Method to Characterize Activated Carbons

**DOI:** 10.3389/fchem.2020.595230

**Published:** 2021-01-28

**Authors:** Jose Carlos Alexandre de Oliveira, Ana Luisa Galdino, Daniel V Gonçalves, Pedro F. G. Silvino, Celio L. Cavalcante, Moises Bastos-Neto, Diana C.S. Azevedo, Sebastiao M. P. Lucena

**Affiliations:** Departamento de Engenharia Química, Campus do Pici, Universidade Federal do Ceará, Fortaleza – CE, Brasil

**Keywords:** adsorption, activated carbon, molecular simulation, PSD, characterization

## Abstract

We propose a pore size analysis methodology for carbonaceous materials that reduces complexity while maintaining the significant elements of the structure-property relationship. This method chooses a limited number of representative pores, which will constitute a simplified kernel to describe the pore size distribution (PSD) of an activated carbon. In this study we use the representative pore sizes of 7.0, 8.9, 18.5, and 27.9 Å and N_2_ isotherms at 77.4 K to determine the PSD which is later applied to predict the adsorption equilibrium of other gases. In this study we demonstrate the ability to predict adsorption of different gas molecules on activated carbon from the PSD generated with representative pores (PSD_rep_). The methodology allows quick solutions for large-scale calculations for carbonaceous materials screening, in addition to make accessible an easily understood and prompt evaluation of the structure-property relationship of activated carbons. In addition to the details of the methodology already tested in different fields of application of carbonaceous materials, we present a new application related to the removal of organic contaminants in dilute aqueous solutions.

## Introduction

Activated carbons are disordered solids made up mainly of carbon, with a high degree of porosity and a high internal surface, giving them great versatility in applications ranging from adsorption to catalysis (Bansal and Goyal, 2005). Their properties in such applications depend largely on the geometry, volume and size of the pores, information that is condensed on the pore size distribution function (PSD) (Jaroniec et al., 1989). The current method of calculating the pore size distribution of the activated carbons is based on the linear combination of simulated isotherms in pores of defined sizes and shapes, obtained through molecular simulation methods (Seaton et al., 1989; Cracknell et al., 1993; Neimark et al., 2009). An ordinary PSD is based on kernels with 200–300 individual pore sizes from which 15 to 20 different pores sizes are chosen to characterize a particular activated carbon. This complex representation makes it difficult to interpret important nuances between the various porosity ranges for users that are not specialized with characterization methods. On the other hand, the ultra-simplified representation of assigning a single pore size to the activated carbon still persists in the literature and in commercial analytic equipments. Such simplification persists because the one pore carbon representation is more easily understandable.

We propose that it may be possible to define an intermediate pore representation between those two extremes that can carry important information from the activated carbon microstructure so that it is quickly understood by a non-specialist. This representation would also benefit the studies for adsorption prediction based on molecular simulation associated with numerical modeling of PSA (pressure swing adsorption) industrial units. We know that it is not possible to experimentally test the wide diversity of carbonaceous materials for a given application. To deal with this time intensive and costly task, numerical methods using two scales may be combined: molecular simulation to predict isotherms and numerical simulation to design PSA units that involve multicomponent gas mixtures. Cases of multiscale material screening involving separation of hydrocarbon mixtures in MOFs and of CO_2_/N_2_ in 13X zeolite have recently been published (Zhang et al., 2015a; Wu et al., 2017; Farmahini et al., 2018). In order to predict the adsorption of multicomponent mixtures on activated carbons, specific kernels for each gas species need to be built. The more pores in the PSD, the more exhaustive are the calculations and complexity to deal with the data for obtaining the isotherms. A method based on certain representative pores may save considerable time in the reproduction of activated carbon adsorption isotherms associated or not with numerical methods for adsorption units design.

Our proposal is to reduce the kernel to three or four pore sizes carefully chosen in order to represent the different regimes of local isotherms filling in the activated carbon. In addition to allowing large-scale calculations for carbonaceous materials screening, this representation carries more meaning and is more easily understood by non-specialists. This methodology has been successfully applied to different activated carbons in determining the adsorption capacity of hydrocarbons, H_2_S and dyes (Lucena et al., 2013; Aguiar et al., 2016; Gonçalves et al., 2018). This study then aims to present the details of the methodology that has been developed in the last years and to present new application in diluted aqueous solutions. Thus, in unit 2, the principles of the methodology and procedures of kernel creation are discussed with special detail for the treatment necessary to obtain significant isotherms for each pore size. In unit 3, the results already obtained and new results are discussed. Since this methodology could be easily implemented in existing analytic equipments, we believe that an immediate gain in interpretation of the microstructure-function relationship would be obtained. Besides that, reducing the complexity of multiscale calculations shall benefit all studies involving the applications of activated carbons for adsorption separations.

## Method and Models

### Representative Pores

**FIGURE 1:**
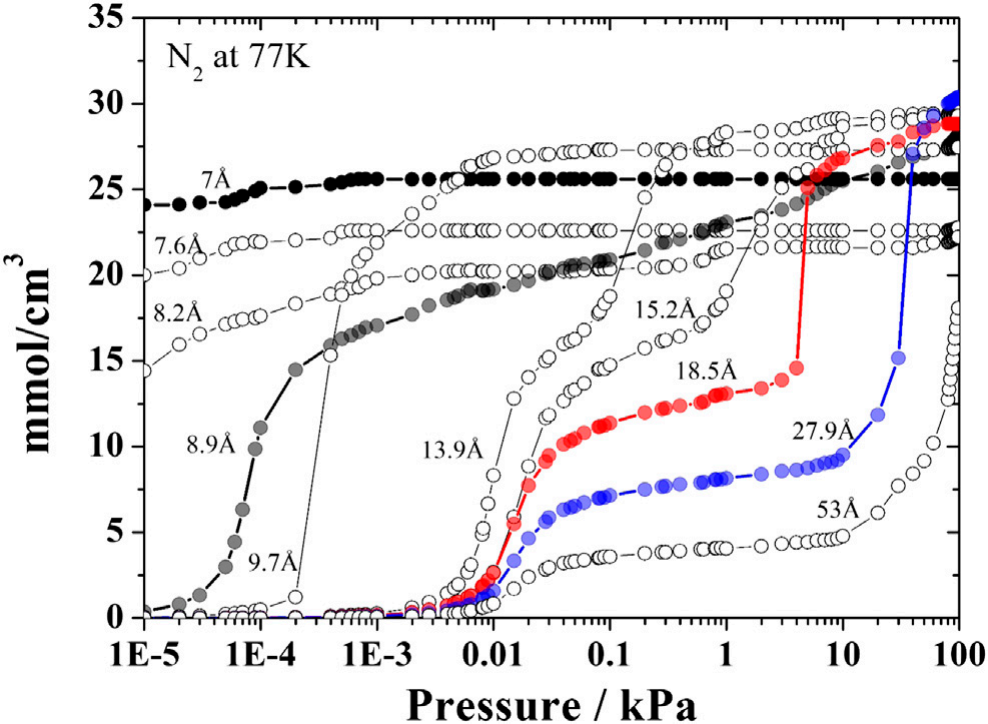
Selected pores of the kernel of N_2_ isotherms obtained at 77.4 K with the representative pore isotherms (7, 8.9, 18.5, and 27.9 Å) highlighted with different colors.

Three pore filling regimes can be identified in a typical kernel of adsorption isotherms. In the first regime, the pore fills continuously and abruptly a monolayer is formed in the central region corresponding to the solid-gas potential well. This occurs in smaller pores and the 8.9 Å pore was defined as representative of this regime. The 18.5 Å pore represents the second filling regime in which the formation of two well-defined layers is observed. For pore sizes of 27.9 Å and higher, multilayer adsorption occurs. Thus, the pores of 8.9, 18.5, and 27.9 Å sizes represent the different filling regimes that can be found for the probe gases regularly employed to characterize activated carbons. In Figure 1 we highlight the pores chosen for nitrogen at 77.4 K. The number of layers of adsorbed gas formed in each pore size generates the particular characteristics of the adsorption isotherms. After establishing the three pore sizes, we noticed that for highly microporous carbons, where there is a significant volume of pores in the ultramicroporous range (pores <7 Å), it was necessary to include another pore size to represent this ultramicroporous volume, usually evaluated using CO_2_ adsorption at 273 K. Thus, a 7Å pore size was added to represent this ultramicroporus volume. Thus, in cases where the adsorbate molecule has a compatible size and the activated carbon has pores in this range, the 7Å pore must be included to the set of representative pores (Gonçalves et al., 2018). Again, Figure 1 may be used to illustrate the particular behavior of isotherms in the ultramicroporous range; for very low pressures these pores are already practically completely filled. It is important to note that this condensation of the kernel in a smaller number of pores does not cause significant loss of information in the recovery of the experimental isotherm with the advantages already previously mentioned.

Our representative pore kernel is based in standard nitrogen isotherms at 77.4 K. Nitrogen isotherms dominate the literature of activated carbon studies thus it is important to use this broad database. Even though shortcomings of the slit-pore model and gas molecule models related to networking effect and adsorbate—carbon interactions have been discussed (Quirke and Tennison, 1996; Davies and Seaton, 1999; Lucena et al., 2020), the predictive ability of an N_2_-isotherm PSD can be maximized through careful validation of the parameters of intermolecular interactions (Ravikovitch et al., 2000).

### Pore Size Distribution

The model used for local isotherms assumes that the complex internal structure of the carbon can be described in terms of a collection of pores of a certain shape or geometry, which adsorb the gas molecules independently of each other. The hypothesis of independence between the pores means that adsorption is carried out in each pore individually, regardless of what happens to the other pores. Under this hypothesis and assuming the same geometry for all pores, the link between the experimental measurements and the model is given by the following integral equation:

$$N\left(Pi,T\right)≅\int_{H_{\min}}^{H_{\max}}\rho \left(H,Pi,T\right)\;f\left(H\right)dH$$ (1)

where *N*(*P*_*i*_ *,T*) is the experimental isotherm expressed as the total amount of adsorbate per Gram of adsorbent at pressure *Pi*; *ρ(H,Pi,T)* represents the simulated local isotherms database (Kernel), obtained for pores with different sizes (*H*), expressed as total adsorbate uptake at pressure *p* per pore volume and *f(H)* represents the PSD. The PSD is computationally determined through the deconvolution of the kernel of simulated isotherms using an experimental probe-gas isotherm. The solution consists of the best combination of local isotherms that would reproduce the experimental isotherm (Davies et al., 1999).

For clarity we named “full” PSD (fPSD) when the pore size distribution is obtained from a complete kernel of isotherms and “representative” PSD (PSD_rep_) for kernel with only representative pores. The characterization was done with the kernel N_2_ at 77 K.

To calculate the PSD_rep_, we now assume that our set of local isotherms that can describe the solid is composed of only three or four isotherms, obtained from the representative pores. As in fPSD, the link between the experimental measurements and the model is given by the same integral Eq. 1. Once our PSD_rep_ is determined, it is possible to predict the theoretical isotherm (Ntheorical) or the maximum theoretical capacity (Qmax) of any component for each carbon. N_theorical_ is estimated based on the PSD_rep_ and the simulated adsorbed quantity of each component (Eq. 2).

$$N_{theorical}\left(Pi,T\right)≅\int_{H_{\min}}^{H_{\max}}\rho^{i}\left(H,Pi,T\right)\;f_{RP}\left(H\right)dH$$ (2)

The *ρ* ^*i*^(*H,Pi,T*) represents the simulated local isotherms database for component i (dye, H_2_S, hydrocarbons, etc.) and *f*_*RP*_(*H*) represents the PSD_rep_. Qmax is determined by the sum of the products of the volume of each pore (Vp_n_) by the maximum simulated quantity (qmax_n_) in each pore (Eq. 3).

$$Q_{\max}=\sum_{n=1}^{N}V_{p_{n}}\times q_{\max_{n}}$$ (3)

### Models

#### Carbon Model

The slit-pore model was used to represent the carbons. Three sheets of graphene with an interlayer space of 3.5 Å were applied to compose each pore wall. The accessible volume for each pore size was calculated using Zeo++ software (Willems et al., 2012) and a probe sphere of r = 1.805 Å. Proper solid-solid parameters were used for each adsorbate molecule and are presented in Table 1. These forcefield parameters have been previously validated in some earlier studies (Lucena et al., 2010; Lucena et al., 2013; Aguiar et al., 2016; Gonçalves et al., 2018).

**TABLE 1:** Solid-solid forcefield parameters applied for each adsorbate molecule.

**Adsorbate molecule**	**σ, Å**	**ε/k**_**B**_ **, K**	**References**
N_2_	3.37	31.3	A
Alkanes	3.4	24.6	B
H_2_S		37	C
Dyes		28	D

A, Lucena (Lucena et al., 2010); B, Lucena (Lucena et al., 2013); C, Gonçalves (Gonçalves et al., 2018); D, Aguiar (Aguiar et al., 2016). Phenol see Kowalczy (Kowalczyk et al., 2018).

#### Molecular Models

We applied the same molecular models that were used to validate the solid-fluid interaction previously. Nitrogen molecules were modeled as a single-site atom with forcefield parameters taken from Ravikovitch et al. (Ravikovitch et al., 2000). TraPPE-UA forcefield (Martin and Siepmann, 1998) parameters were used for the alkane molecules. The H_2_S molecule was represented by the model proposed by (Kristóf and Liszi, 1997). Parameters for dye molecules were taken from universal forcefield (Rappe et al., 1992). The atom-atom model was used for the Phenol molecule as suggested by Kowalczyk (Kowalczyk et al., 2018).

### Simulation Details

The simulation of adsorption isotherms in the slit pore model has been investigated using the Grand Canonical Monte Carlo (GCMC) method because it allows a direct calculation of the phase equilibrium between a gas phase and an adsorbed phase. The 12–6 Lennard-Jones potential was used to describe the fluid-fluid and solid-fluid interactions.

$$U\left(r_{ij}\right)=4\varepsilon_{ij}\left[{\left(\frac{\sigma_{ij}}{r_{ij}}\right)}^{12}-{\left(\frac{\sigma_{ij}}{r_{ij}}\right)}^{6}\right]$$ (4)

Here, ε_ij_ is the well depth, σ_ij_ is the molecule diameter, and r_ij_ is the distance between the interacting atoms *i* and *j*. The cross terms were obtained through Lorentz-Berthelot arithmetic and geometric combination rules applied in most of the named class I forcefields (Harrison et al., 2018). The values of the parameters included in the interaction Lennard Jones potentials are given in Table 1. The Monte Carlo computations were done in the RASPA code (Dubbeldam et al., 2016) and the slit-pore model was built with an in house application. At least 10^5^ Monte Carlo cycles were performed for equilibration and an additional 10^5^ cycles in the production phase, where the properties were averaged. Each cycle contains N steps, where N is the number of guest molecules in the system. The potential cut-off distance was 13 Å as validate by studies in similar systems (Martin and Siepmann, 1998; Ravikovitch et al., 2000; Lucena et al., 2013). Simulation cells were replicated to at least 26 Å along each axis, for the minimum image convention to be satisfied. Absolute adsorbed amount (simulated) was converted into excess adsorbed amount via Eq. 6:

$$N_{exc}=N_{abs}-V_{p}\rho_{g}$$ (5)

where Nabs is the absolute amount, Nexc is the excess amount, Vp is the accessible pore volume and ρg is the gas-phase density estimated by Peng-Robinson equation.

## Results and Discussion

### Performance of the PSD_rep_ Kernel

An approach to evaluate how much information would be lost when the PSD is reduced to a limited number of representative pores would be to compare the ability of the PSD_rep_ to represent the original experimental N_2_ equilibrium isotherm at 77 K. This check was performed for data relative to 3 different commercial activated carbon samples (BPL, WV1050, Maxsorb), previously reported (Lucena et al., 2013). These carbons were chosen to intentionally cover different volumes and microporosities usually observed in commercial activated carbons. BPL has the lowest volume of micropores in the series with a total pore volume (Vp) of 0.59 cm^3^/g. The WV1050 carbon has an intermediate volume (Vp = 1.07 cm^3^/g) while Maxsorb (Vp = 1.56 cm^3^/g) is classified as an ultra-microporous carbon.

**FIGURE 2:**
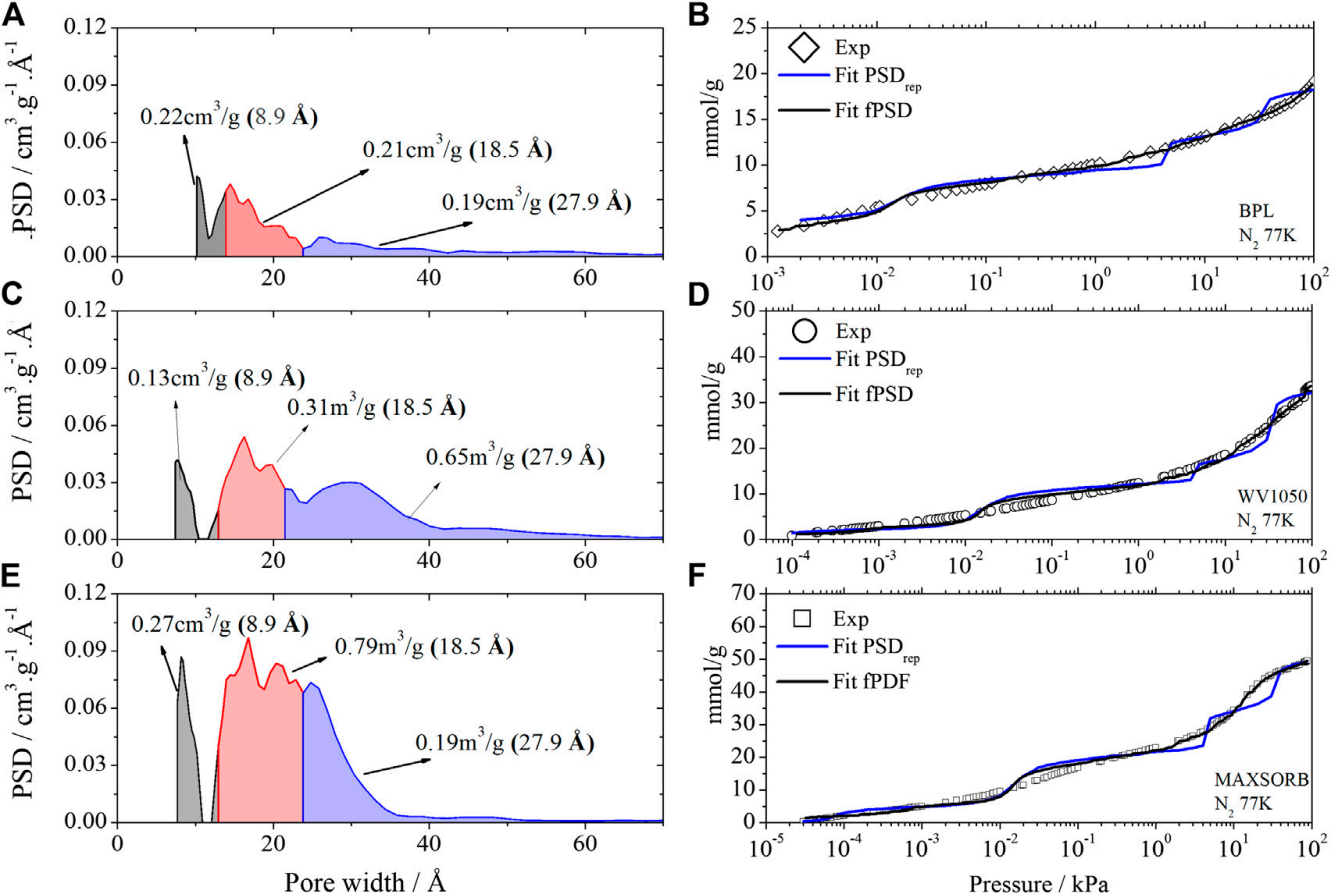
PSD_**rep**_ of BPL **(A)**, WV1050 **(C)** and Maxsorb **(E)** activated carbons calculated from the kernel A and Kernel A1 of the N_2_ isotherms obtained at 77.4 K. Comparison of isotherm fits using fPSD and PSD_rep_ for BPL **(B)**, WV1050 **(D)** and Maxsorb **(F)**. Gray color – total volume of the 8.9 Å pore. Red color – total volume of the 18.5 Å pore. Blue color – total volume of the 27.9 Å pore.

Using the kernel that is available in analytical equipments which consists of ca. 300 isotherms, we estimated the full Pore Size Distribution (fPSD) and the respective simulated N_2_ isotherms (see Figure 2) for those three commercial carbon samples. By applying again the same algorithm, but now using only the three representative pores as proposed in this study (8.9, 18.5 and 27.9 Å), we may estimate the PSD_rep_ and their respective simulated N_2_ isotherms, as also shown in Figure 2.

It may be observed that the proposed PSD_rep_, using only the 3 representative pores isotherms, represents satisfactorily the experimental N_2_ isotherm, especially when compared to the representation of the full PSD, obtained with a kernel of 300 isotherms. In general it should be noted the excellent agreement at low pressures (<1 kPa) and the appearance of some “S curve” deviations at higher pressures.

In addition, some textural properties of carbon samples used in previous representative pore studies using both fPSD and PSD_rep_ are presented in Table 2, indicating that in general the pore volumes are consistently well represented by the proposed PSD_rep_ method.

**TABLE 2:** Total area, total volume obtained from molecular simulation for complete NLDFT kernel (fPSD) and with representative pores (PSD_rep_).

Sample	fPSD (NLDFT)		PSDrep	
	Area (m^2^/g)	V_total_ (cm^3^/g)	Area (m^2^/g)	V_total_ (cm^3^/g)
Noris RB4	907	0.50	761	0.533
WV1050	1258	1.071	1079	1.10
Maxsorb	2305	1.564	1915	1.69
BPL	972	0.597	862	0.63
PC12	727	0.241	579	0.2890
PC35	1355	0.543	1066	0.6160
PC58	1599	0.826	1309	0.9190

To better illustrate how the proposed PSD_rep_ may assist in quicker analysis and correlation of carbon structures and properties, we present in Table 3 the calculated pore volumes of the 3 carbon samples used for this study for each of the representative pores that were chosen. According to IUPAC (Thommes et al., 2015), micropores are in the range 0–20 Å, so in this interval we have the information of the lower size pores associated to the 8.9 Å pore, and, simultaneously, the higher size pores represented by the 18.5 Å pore. These two limits in pore sizes within the micropore region have significant differences in their filling mechanisms, so the separate identification of each volume (low and high pore sizes) improves the thus obtained PSD information. In sequence, we have the pore size range associated to the 27.9 Å, related to the mesoporosity. This range around 27.9 Å pore is usually more significant for the adsorption process, since for pores above 70 Å the molecules would adsorb more freely on the carbon surface with negligible wall effects.

**TABLE 3:** Pore volume correlated with characteristic pores for each activated carbon.

**Pore size (Å)**	**BPL (cm** ^**3**^ **/g)**	**WV1050 (cm** ^**3**^ **/g)**	**Maxsorb (cm** ^**3**^ **/g)**
8.9	0.24	0.12	0.27
18.5	0.23	0.31	0.79
27.9	0.11	0.64	0.63
**Total**	**0.58**	**1.07**	**1.69**

The bold entries correspond to the total volume of each sample.

From the distribution of individual pore volumes among the different pore sizes, we may observe that BPL has the largest micropores/total pores volume ratio, i.e., 81% of its total pore volume is almost evenly distributed between both lower and higher range of micropore sizes (8.9 and 18.5 Å). On the other hand, WV1050 presents the highest mesoporosity (27.9 Å) volume ratio, *c. a. *60% and Maxsorb has the highest concentration of micropores in the higher size range (18.5 Å), nearly 47%. For the absolute pore volume values, we observe that Maxsorb has higher values than the other two carbon samples in all individual pore sizes. These important remarks are immediately obtained from a quick evaluation of the pore volumes data as presented in Table 3.

In the following section we will attempt to associate several structure-properties correlations to the volumes associated with each pore size using the PSD_rep_ method.

### Specific Applications Using the PSD_rep_ Method for Activated Carbons

#### Hydrocarbons

We have used the Representative Pores PSD method (PSD_rep_) to obtain adsorption data of C1-C4 mixtures in natural gas storage reservoirs (Lucena et al., 2013; Mileo et al., 2014). This problem involves the calculation of multicomponent mixtures at each pore size to estimate the amount adsorbed in each activated carbon sample. If one uses a usual PSD with 20 pore sizes, the calculation effort would be huge. In this case, the representative pores approach can reduce significantly the simulations time keeping nearly the same results in obtaining the adsorption isotherms of the C1-C4 hydrocarbons.

**FIGURE 3:**
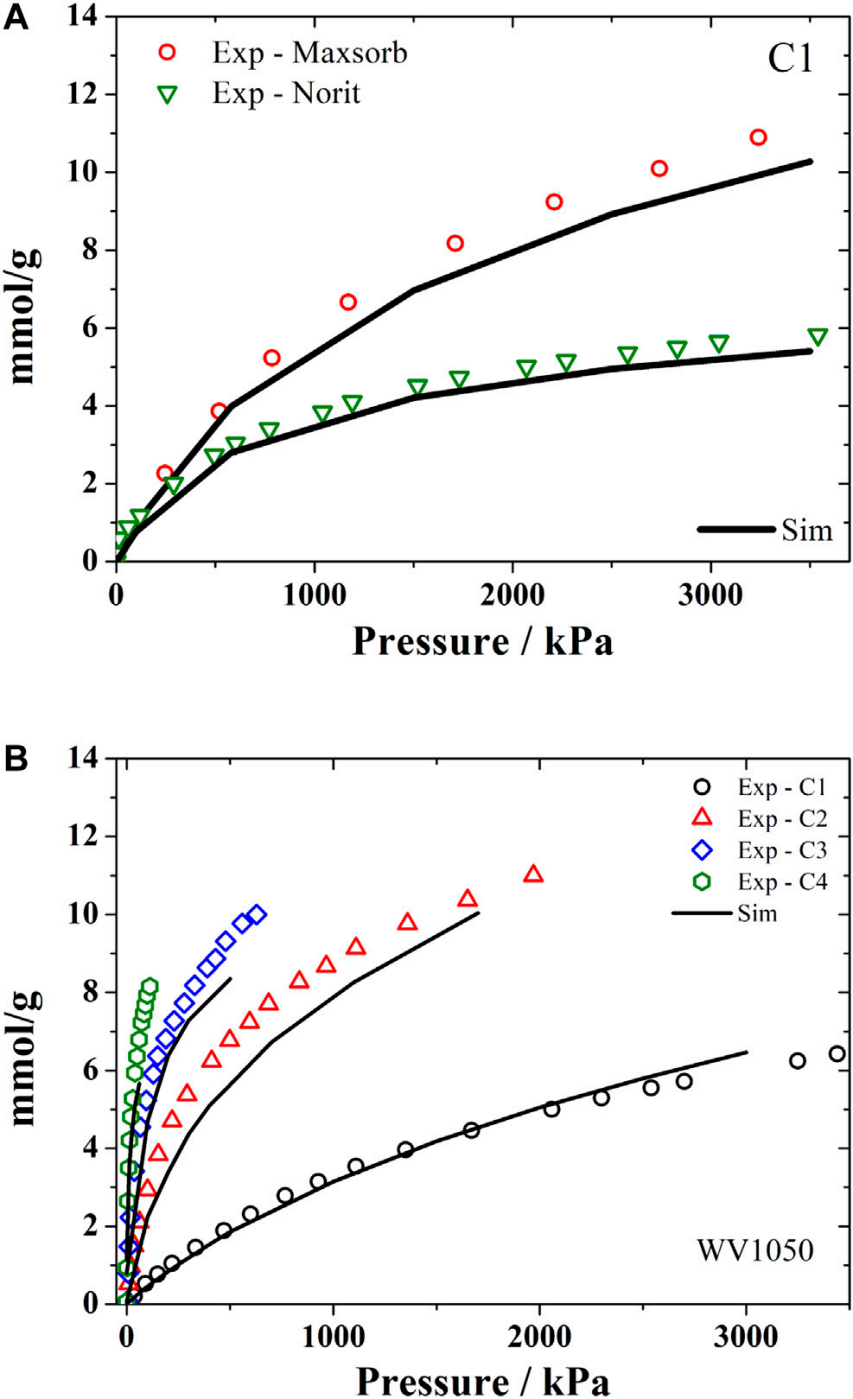
Experimental and simulated (using PSD_rep_) adsorption monocomponent isotherms of hydrocarbons. **(A)** methane at 298 K in Maxsorb and Noris R1; and **(B)** alkanes C1-C4 at 298 K in WV1050.

As seen in Figure 3, there is a very good agreement of the experimental isotherms for methane in Maxsorb and Norit R1 samples (Figure 3A) and for all C1-C4 alkanes in WV1050 (Figure 3B) with the simulated isotherms obtained using the PSD_rep_ method. These simulation results could then be applied to predict the long term deactivation of the natural gas storage systems by the heavier alkanes (C3 and C4) (Lucena et al., 2013) in agreement with available experimental data (Pupier et al., 2005; Walton and LeVan, 2006; Rios et al., 2011). In this case the shortened simulation times are very helpful since experimental results for long term deactivation in natural gas storage systems are particularly laborious and therefore difficult to find in the open literature. Thus the adsorbent selection step for these systems would become considerably abbreviated when using this methodology of representative pores PSD.

We have also applied the PSD_rep_ method to integrate the scale of molecular simulation with the numerical formulation used in designing adsorption processes. Since the calculations used for the design of those processes is heavily based on the experimental data of the adsorption equilibrium between the sorbates and the sorbents, our approach intended to reduce the experimental effort for a suitable process design by using this representative pores (PSD_rep_) method to estimate the equilibrium isotherms needed. So somewhat more complex mixtures, such as C1-C5, or including eventual contaminants like CO_2_ and N_2_, have been evaluated using PSD_rep_ (Submitted). Also, the results of an earlier reported experimental study by Pupier (Pupier et al., 2005) have been very satisfactorily reproduced using only the simulated isotherms from the PSD_rep_ method.

In both cases, it became clear that deactivation of the natural gas storage system is minimized when the carbon pore size fraction of 18.5 Å is maximized.

#### H_2_S

The removal of hydrogen sulfide (H_2_S) from industrial process streams has received wide attention recently due to its high toxicity and corrosive potential to damage industrial piping and equipments (Zhang et al., 2015b). For this study, a more refined evaluation of the size pores distribution was definitely needed, since H_2_S adsorption occurs mostly in ultramicropores (<7 Å) at very low pressures. This was indeed a common characteristic of several activated carbon samples, chemically impregnated, that were studied for the removal of H_2_S: all of them presented high ultramicroporosity volumes. The volume of ultramicropores had to be experimentally evaluated using CO_2_ at 273 K as probe molecule, since N_2_ at 77 K is not able to access this low range of pore sizes (Rouquerol et al., 2014).

**FIGURE 4:**
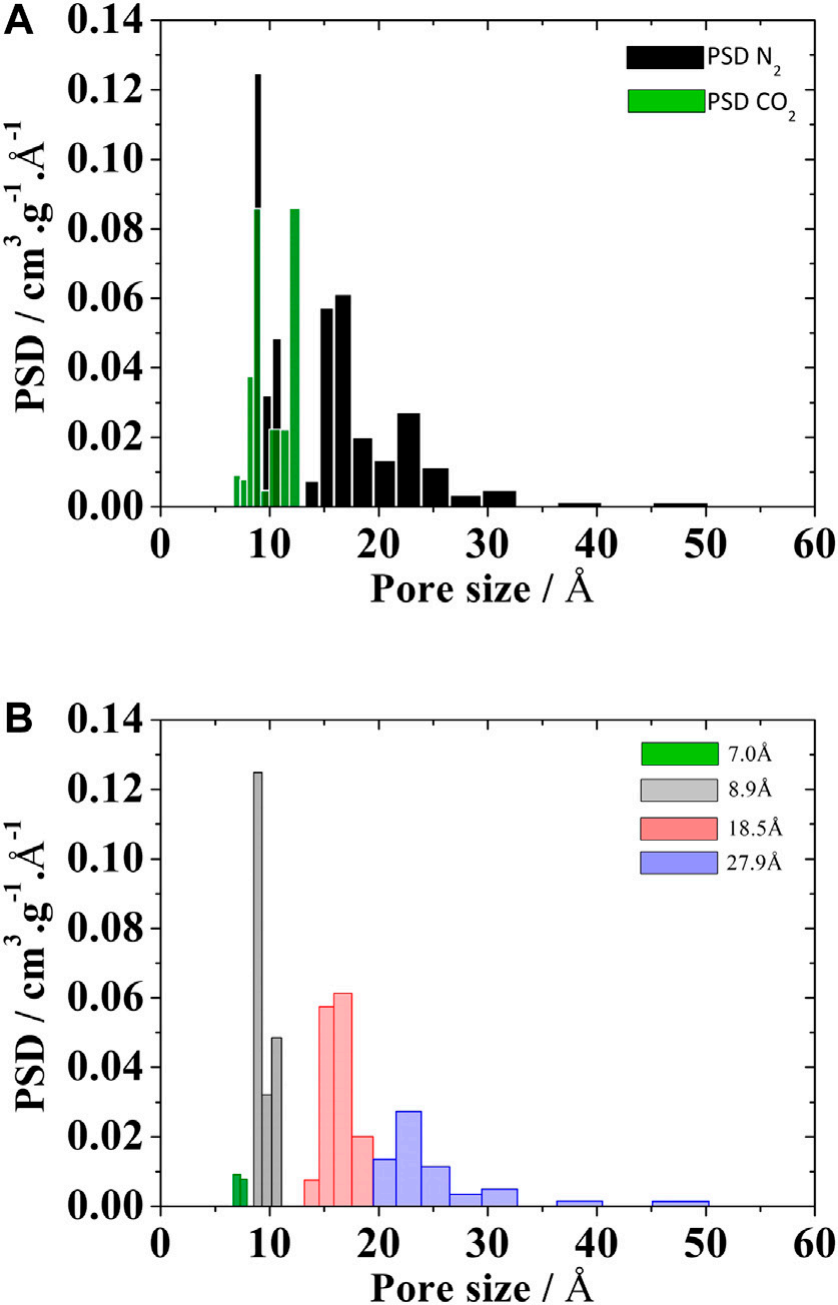
PSD of Noris RB4 activated carbons calculated from the **(A)** kernel of CO_2_ at 273 K e N2 at 77.4 K and **(B)** PSD_rep_ from kernel of the N2 at 77.4 K plus CO_2_ volume of the 7Å pore.

Using the representative pores methodology, the PSD_rep_ of Norit RB4 carbon was obtained including three pores (8.9, 18.5 and 27.9 Å) from the N_2_ isotherm at 77.4K, plus one pore (7 Å) that incorporated the total ultramicropore volume, measured by CO_2_ adsorption at 273 K, as presented in Figure 4.

**FIGURE 5:**
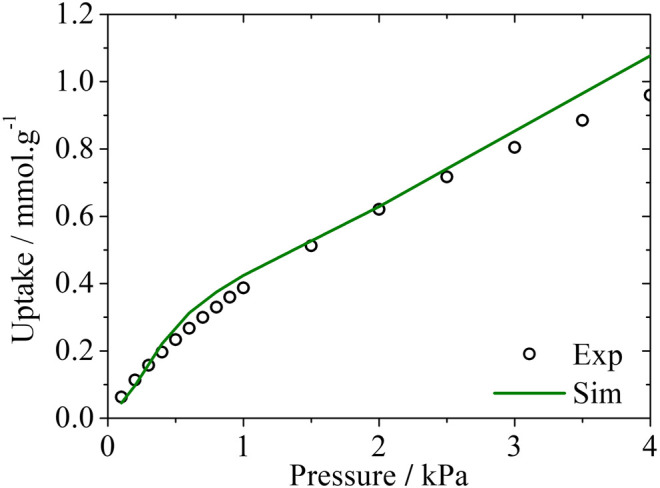
Experimental and simulated adsorption isotherms for H_2_S in Noris RB4 at 293 K. Experimental data taken from Cruz (Cruz et al., 2005).

The experimental isotherm of H_2_S in that carbon sample is shown in Figure 5, along with the simulated isotherm calculated using the PSD_rep_ method.

The very good agreement between the H_2_S experimental and simulated adsorption data is a promising insight for future studies of H_2_S adsorption in activated carbons and further validation for the representative pores method.

#### Dye

**FIGURE 6:**
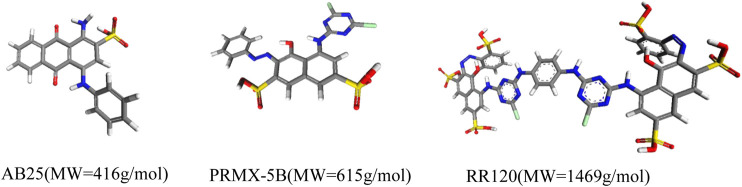
Dye molecules and respective molecular weights.

The textile industry has faced a serious problem of contamination of effluents by dyes (Órfão et al., 2006). One of the treatment alternatives has been the use of activated carbons. This is another opportunity to apply the representative pore methodology to predict which carbon would be most suitable for removing a particular dye (Namasivayam and Kavitha, 2002). We carried out a study (Aguiar et al., 2016) showing the correlation of the representative pores with the adsorption of dyes for the activated carbon Norit R1, WV1050 and Maxsorb and the dyes Acid Blue 25; Procion Red MX5B and Reactive Red 120, with increasing sizes (Figure 6).

Simulations in the NVT ensemble in the 3 representative pores showed that Maxsorb carbon would have the highest adsorption capacity, which was proven in the experiments. Another interesting information concerns the impossibility of the RR120 dye to access the 8.9 Å pores, meaning that activated carbons designed for this dye should minimize this pore range. Comparing the experimental results with the simulated results it was also possible to identify effects of molecule-molecule interaction. Contrary to what the simulation predicted; the three carbons tested adsorbed equal amounts of RR120. This would be possible if the dye RR120 only adsorbed superficially. The molecular simulation in the representative pores showed that if the RR120 dye formed dimers, in addition to the 8.9 pore, already inaccessible for individual molecules, the remaining pores would no longer be compatible (Table 4). The analysis allowed us to conclude that conventional activated carbons are not suitable adsorbents for molecules with dimensions of the order of magnitude that of the dye RR120.

**TABLE 4:** Pore filling in number of molecules predicted by molecular simulation in the NVT ensemble.

**Dye molecule**	**Pores**			**Loading Steps**
	8.9 Å	18.5 Å	27.9 Å	
**AB25**	2	7	13	4 x 10^6^
**PRMX-5B**	1	5	10	4 x 10^6^
**RR120**	0	1	3	4 x 10^6^
**RR120 dimer**	0	0	0	6 x 10^6^

### Diluted Solutions

The prediction of contaminants adsorption removal (at low concentrations) from aqueous solutions represents a formidable challenge for molecular simulation. Depending on the nature of the contaminant molecule present in the solution, there might be no reliable vapor pressure data to apply the Monte Carlo method in the grand canonical ensemble. On the other hand, the very low concentration of the solute prevents the use of molecular dynamics since this would require the construction of simulation boxes above the current computer processing capacity.

**FIGURE 7:**
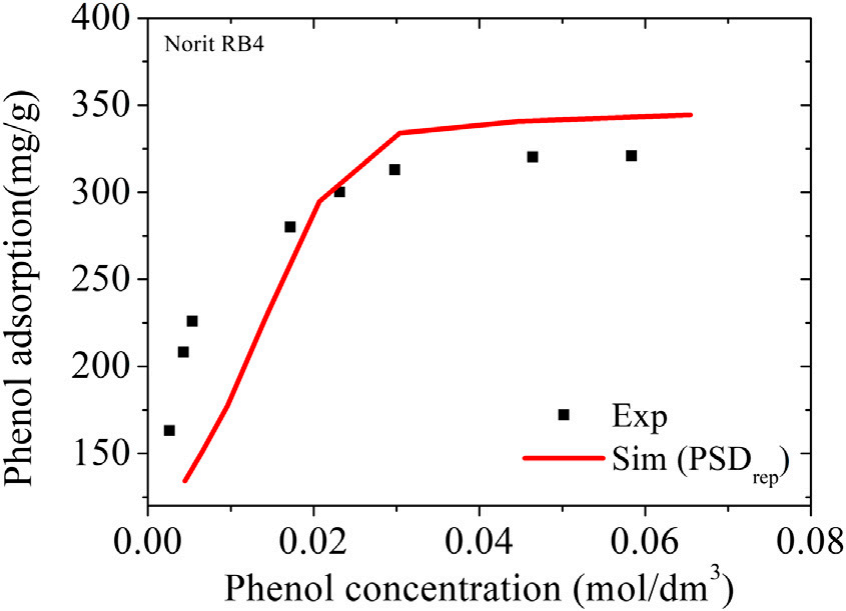
Adsorption of phenol in NCB-8h carbon. Symbols are experimental data at 298 K from Kowalczyk (Kowalczyk et al., 2018); the line represents the simulation results using PSD_rep_ from Noris RB4 with total pore volume adjustment by 18%.

A recent study by Kowalczyk (Kowalczyk et al., 2018) is an opportunity to apply the representative pores method in diluted phenol solutions. In that study, the authors concluded that the micropores of the tested activated carbons at saturation and 301 K adsorb only phenol with the activated carbons showing selective molecular sieving effects with respect to water. If this prevailing molecular sieving effect is generalized to other solute concentrations, the calculation of the adsorption isotherm could be done using the grand canonical ensemble with the fugacity of the gas phase corresponding to the fugacity of phenol in equilibrium with the diluted solution (Chempath et al., 2004). Because the solution is very diluted, the fugacity cannot be calculated using equations of state. In this case, it is recommended to obtain the activity coefficient of phenol in the solution at the specific experimental conditions to estimate the value of the Henry’s Law constant (HLC). We selected the activity parameters found by Janini and Qaddora (Janini and Qaddora, 1986) obtained by liquid-liquid chromatographic processes with underlying phenomenology similar to the adsorption of phenols on activated carbons. Using the equation:

$$P_{i,G}=H_{pc}*\left[C_{i,L}\right]$$ (6)

where *C*_*i,L*_ and *P*_*i,G*_ are the concentrations of the solute in the liquid and in the gas phases, respectively, and *H*_*pc*_ is the Henry’s Law constant (HLC) in kPa/mol.L. With an estimated value for H_pc_ = 0.61, it was possible to calculate the adsorbed amounts of phenol for the entire concentration range using the Norit RB4 carbon (area = 907 m^2^/g; V = 0.50 cm^3^/g). This carbon has textural properties very similar to the NCB-8h carbon (area = 1186 m^2^/g; V = 0.41 cm^3^/g) used in that experiment. The total volume of Norit RB4 carbon obtained by the representative pores method was reduced by 18% to match the total volume of the NCB-8h carbon. The complete simulated isotherm thus calculated is compared to the experimental data in Figure 7.

**FIGURE 8:**
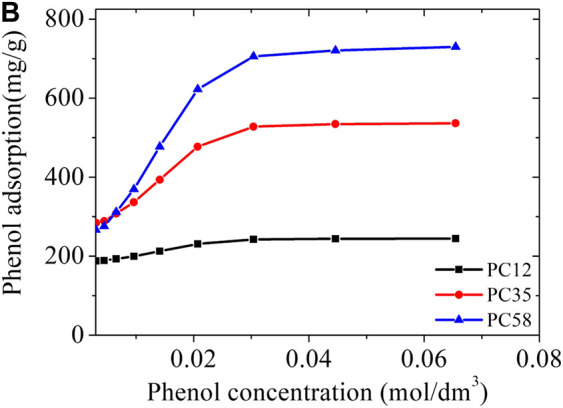
Predicted phenol adsorption isotherms in a PC series of activated carbons at 298 K.

The value found with the PSD_rep_ at the adsorption saturation (301 K and 0.059 kPa) was 349 mg/g, practically the same as the theoretical value of 350 mg/g estimated by Kowalczyk (Kowalczyk et al., 2018) from a 23-pore PSD in the range 7–23 Å. In addition, the complete simulated isotherm closely follows the experimental values for the NCB-8h sample, indicating that the use of the Henry´s Law Constant may be an effective tool for estimating adsorption isotherms based on molecular simulations in diluted aqueous solutions.

To demonstrate the versatility of the representative pores method, we applied it to a series of carbons PC58 to PC12, neutral and without species that promote hydrophilicity, also made with polymer precursor (PET) similar to the material used in the synthesis of NCB-8h (styrene-divinylbenzene copolymer) Kowalczyk (Kowalczyk et al., 2018) and quickly estimated their adsorption isotherms for phenol at 298 K (Figure 8).

The PC58 sample was predicted to have a phenol adsorption capacity of 743 mg/g, twice the capacity of the material tested by Kowalczyk (Kowalczyk et al., 2018). This high adsorption capacity of the PC58 sample is clearly related to the total pore volume which is approximately double that of the NCB-8h sample (see Table 5). An attractive feature of PSD_rep_ is that the importance of each porosity range is easily correlated with the application. We observed that the PC35 carbon has a slightly higher microporosity than the PC58 carbon, but what in fact determines the higher adsorption capacity of phenol in PC58 is the mesoporosity region, being *ca.* 50% larger than in PC35.

**TABLE 5:** Pore volumes correlated with representative pores for each activated carbon in the PC series (Parra et al., 2002).

**Pore size (Å)**	**PC12 (cm** ^**3**^ **/g)**	**PC35 (cm** ^**3**^ **/g)**	**PC58 (cm** ^**3**^ **/g)**
8.9	0.23	0.34	0.32
18.5	0.06	0.27	0.44
27.9	0.00	0.00	0.16
**Total**	**0.29**	**0.61**	**0.92**

The bold entries correspond to the total volume of each sample.

## Conclusion

From a reduced kernel of adsorption isotherms composed of representative pores, we determined the Pore Size Distribution (PSD_rep_) of several activated carbons based on the N_2_ isotherm at 77.4 K. Next, some studies on the adsorption of other gases involving hydrocarbons, H_2_S, and phenol in dilute aqueous solution, were presented. The last example represents a new opportunity to apply the methodology of representative pores and, to the best of our knowledge, this is the first time that isotherms of a solute diluted in water are predicted combining the Monte Carlo method in the grand canonical ensemble with the fugacity obtained in the Henry´s Law domain. We believe that the representative pores method may be an interesting approach to reduce the load of numerical calculations in multiscale applications, while making the significant use of information contained in a PSD more accessible to predict the structure-property relationship of activated carbons. An integrated application for determination of PSD_rep_ based on N_2_ adsorption isotherm at 77.4 K can be accessed in our repository at www.lab3d.ufc.br (characterization section).

## Data Availability Statement

The original contributions presented in the study are included in the article/Supplementary Material, further inquiries can be directed to the corresponding authors.

## Author Contributions

All authors assisted in the development and writing of the paper. JO and SL wrote the article. JO, SL, DG, AG, and PS were involved in conceptualization, methodology and doing simulation. MB-N did experiments. CC and DA perform the review and editing of the manuscript.
